# Lemon Juice Formulations Modulate *In Vitro* Digestive Recovery of Spinach Phytochemicals

**DOI:** 10.17113/ftb.60.03.22.7104

**Published:** 2022-09

**Authors:** Valerija Vujčić Bok, Ivana Šola, Gordana Rusak

**Affiliations:** Department of Biology, Faculty of Science, University of Zagreb, Marulićev trg 9a,10000 Zagreb, Croatia

**Keywords:** *Spinacia oleracea* L., antioxidant activity, α-amylase inhibitory activity, bioaccessibility of bioactive compounds, *in vitro* digestion

## Abstract

**Research background:**

*Citrus limon* (L.) Burm lemon juice is rich in many important natural chemical components (flavonoids, citric acid and vitamin C) and its use in traditional medicine is well known. Formulations of lemon juice with fruit polyphenols in beverages have been investigated, but there is very little information about their ability to modulate the digestive behaviour of polyphenols. The goal of this study is to determine the stability and digestive availability of spinach (*Spinacia oleracea* L.) polyphenols by adding different volume fractions of lemon juice (0, 2, 5, 10 and 20%) during *in vitro* digestion.

**Experimental approach:**

The content of polyphenols and other abundant compounds including nitrates, oxalic acid and l-ascorbic acid in spinach formulation with various volume fractions of lemon juice were measured in predigested and digested samples using *in vitro* human digestion model. Antioxidant and α-amylase inhibitory activities of spinach lemon juice formulation were also measured.

**Results and conclusions:**

The highest increases in total polyphenols, total flavonoids, total phenolic acids, oxalic acid and nitrate content were noted in predigested and almost all digested spinach samples formulated with the highest volume fraction of lemon juice. In the same sample, the content of individual compounds significantly increased after salivary  (l-ascorbic acid), initial (*p*-coumaric acid) and intestinal (quercetin) phase of digestion. High bioaccessibility of polyphenols and l-ascorbic acid in all phases of digestion was observed in almost all spinach lemon juice formulations, with the exception of nitrates in gastric and intestinal phases and oxalic acid in the intestinal phase, which had moderate bioaccessibility.

**Novelty and scientific contribution:**

For the first time the stability and digestive availability of spinach polyphenols, oxalic acid, nitrates and l-ascorbic acid were tested with the addition of different volume fractions of lemon juice. The pH of lemon juice and its  l-ascorbic acid content increase the stability and availability of polyphenols in spinach lemon juice formulation during *in vitro* digestion. Antioxidant and α-amylase inhibitory activities increase in dose-dependent manner after lemon juice addition. Accordingly, spinach formulated with 20% of lemon juice appears as the best source of dietary polyphenols with antioxidant and antidiabetic activities and nitrates that may be used as a functional drink.

## INTRODUCTION

Spinach (*Spinacia oleracea* L.) is a functional food that is consumed in fresh (*e.g.* salads and smoothies), cooked (*e.g.* steamed, in soups or in fresh pasta) and dried forms (*e.g.* smoothies) due to its diverse nutritional and chemical composition. It is a good source of minerals (magnesium, potassium and iron), vitamins (K, A, folate and C), carotenoids  (β-carotene, lutein and zeaxanthine) and polyphenols (flavonoids and phenolic acids) (*1*-*3*). So far, various functional properties of spinach leaves and its preparations (extracts, fractions) have been studied, among which are antioxidant, anti-inflammatory, antiproliferative, antiobesity, hypoglycaemic and lipid-lowering activities (*2*). Spinach is also rich in oxalic acid and dietary nitrates (*4**,**5*). Nitrate-rich spinach can promote nitric oxide production, enhance endothelial function and lower acutely high blood pressure. These outcomes may benefit cardiovascular health (*6*) and exercise performance (*7*). Also diet with natural nitrites and nitrates can reduce triglyceride content in blood (*8*). According to Zhang *et al.* (*9*) nutritive value of spinach in diet is limited by its high oxalate content. High oxalate content in diet can disturb bioavailability of some minerals in the intestine and cause deficiency of calcium, iron, magnesium and copper. Also, consumption of additional oxalate can increase the risk of kidney stone formation by inducing a significant increase in urinary oxalate excretion (*9*). Therefore, the measurement of oxalic acid content along with other bioactive compounds is of great importance when it comes to spinach preparations. Lemon (*Citrus limon* (L.) Burm) juice is also rich in many important natural chemical components (flavonoids, citric acid, vitamin C and minerals, *e.g.* calcium and phosphorus) and its use in traditional medicine is well known to treat scurvy, high blood pressure, common cold and irregular menstruation (*10*). Antioxidant activity of flavonoids and vitamin C is the most important factor for its health-promoting properties (*11*) contributing to the reduction of the symptoms of inflammation and excessive formation of reactive oxygen species and thus reducing the risk of developing cardiovascular diseases, diabetes, obesity and cancer (*10*, *12*). Lemon juice is increasingly being utilized in fruit and vegetable drinks and in fresh brewed tea products to improve organoleptic quality and nutritive value of the drink (*13*, *14*). Although lemon juice formulations with fruit polyphenols in beverage systems have already been investigated (*11*, *13*, *15*), the knowledge about their ability to modulate polyphenol digestive behaviour is scarce. Only Green *et al.* (*14*) studied the effect of citrus juices (grapefruit, lemon, lime and orange) on *in vitro* digestive recovery of green tea polyphenols (catechins) and reported the highest catechin recoveries by lemon juice, followed by orange, lime and grapefruit juice. Digestible vitamins C and D may have a positive effect on polyphenol (flavonoids: catechins and anthocyanins) content and bioavailability (*16*). The stability of the polyphenols may also be affected by the pH of the solution (*17*). The pH values and content of vitamins and polyphenols of different citrus juices depend on the variety, condition of growing, harvesting time, storage and juice processing (*18*-*21*). As the most frequently used citrus in the preparation of formulations with fruits and vegetables, lemon has the lowest pH, while lime, grapefruit and orange all have higher pH (*22*). Orange has the highest content of vitamin C, lemon and grapefruit follow with the similar amount, while lime contains the least vitamin C (*14*). Lemons and oranges have similar total polyphenol content, while lime and grapefruit have similar lower values (*23*).

The goal of this study is to determine the stability and digestive availability of spinach polyphenols in a formulation with various volume fractions of lemon juice (0, 2, 5, 10 and 20%) using *in vitro* digestion model. We used fine dry spinach powder for sample preparation to potentially obtain higher values of bioactive compounds. Fine spinach powder can have significantly higher water-binding ability (*3*) and therefore can have positive impact on the extraction of bioactive compounds (*24*). Spectrophotometric (to determine total phenols, total flavonoids and total phenolic acid) and high-performance liquid chromatography (HPLC, to determine individual phenolic compounds: *p*-coumaric acid, ferulic acid and quercetin) methods were used to determine the amount of polyphenols in predigested and digested spinach lemon juice (0, 2, 5, 10 and 20%) formulations and in pure lemon juice. Mass fraction of vitamin C (l-ascorbic acid) as the most abundant vitamin in lemon juice was measured with HPLC in predigested (0, 2, 5, 10 and 20%) and digested (0 and 20%) spinach lemon juice formulations and in pure lemon juice. Spectrophotometric determination of health-promoting dietary nitrates and potentially harmful oxalic acid was also done in all spinach samples and in lemon juice. Determination and comparison of antioxidant activity of all predigested and digested spinach lemon juice formulations and lemon juice was performed with DPPH (1,1-diphenyl-2-picrylhydrazyl) and FRAP (ferric reducing antioxidant power) methods. α-Amylase inhibitory activity was performed to test the predigested spinach formulation and lemon juice for the potential antidiabetic activity. The pH of lemon juice and all predigested and digested spinach lemon juice formulations was also tested.

## MATERIALS AND METHODS

### Materials and preparation of spinach lemon juice formulations

Enzymes (α-amylase, porcine pepsin, lipase and pancreatin) and bile utilized for *in vitro* digestion and antidiabetic activity (α-amylase) were purchased from Sigma-Aldrich, Merck (St. Louis, MO, USA). The 3,5-dinitrosalicylic acid, salicylic acid, 2,2′-diphenyl-2-picrylhydrazyl hydrate (DPPH), (±)-6-hydroxy-2,5,7,8-tetramethylchromane-2-carboxylic acid (Trolox), l-ascorbic acid and all phenol standards: quercetin, caffeic, gallic, *p*-coumaric, and ferulic acids were purchased from Sigma-Aldrich, Merck. The 2,4,6-Tris(2-pyridyl)-*s*-triazine (TPTZ) was obtained from Acros Organics (New Jersey, NJ, USA). Folin–Ciocalteu reagent and all other chemicals were purchased from Kemika (Zagreb, Croatia). Deionized water was used in all experiments and the solvents and chemicals were of analytical or HPLC grade.

Spinach BIO powder (brand: Chefica, certifications: HR-EKO-05, origin: Germany) was purchased in a local health food store Chefica (Zagreb, Croatia). Spinach powder (1 g) was soaked in 50 mL of deionized boiling water (100 °C) and cooked on a laboratory heater TK 23 (TechnoKartell, Noviglio, Milan, Italy) for 40 min to obtain a concentrated extract of 100 mg/mL. The concentrated extract was filtered through a filter paper (Whatman grade 595, pore size 4−7 μm). Lemons were bought in a local store Konzum (sort: Primofiori, net quantity: 500 g, country of origin: Spain). Lemon juice was squeezed from 500 g of lemons. Spinach lemon juice formulations were prepared by adding filtered (Whatman grade 595) *φ*(lemon juice)=0, 2, 5, 10 and 20% to spinach extract solution (100 mg/mL). Spinach extracts were cooled down to 25 °C under running water before adding the lemon juice. Immediately after preparation of spinach lemon formulations, *in vitro* digestion of samples was performed.

### Model of human in vitro digestion

*In vitro* digestion model was performed according to Šola *et al.* (*25*) with slight modification. First, spinach lemon formulations (0.3 mL) were mixed with 0.3 mL of phosphate buffer (20 mM, pH=7). Modification was made by including a salivary phase and initializing the digestion by the addition of 10 μL of amylase (0.48 mg/mL in 20 mM phosphate buffer, pH=7), followed by the incubation for 5 min at 37 °C in a shaking water bath SW22 (Julabo, Seelbach, Germany) at 150 rpm. A volume of 0.4 mL of porcine pepsin solution (3 mg/mL in 0.1 M HCl) was added and acidified with HCl (0.5 M, pH=2) to simulate the stomach digestion, and incubated in a shaking water bath for 1 h at 37 °C and 150 rpm. Sodium bicarbonate (1 M) was added to adjust the pH to 5.3 and mimicked upper intestinal phase of digestion. After pH adjustment, a volume of 0.9 mL of pancreatic juices (bile acids 2.4 mg/mL, porcine lipase 0.2 mg/mL and pancreatin 0.4 mg/mL in 20 mM phosphate buffer, pH=7) was added. The final volume of each sample, both before and after digestion, was brought to 2 mL with phosphate buffer (20 mM, pH=7). The final pH was adjusted additionally to 7 with 1 M NaOH and then incubated for 2 h at 37 °C in a shaking water bath at 150 rpm. Samples were centrifuged (Hettich MIKRO 220R; Andreas Hettich GmbH & Co., Tuttlingen, Germany) after *in vitro* digestion at 4 °C and 9469×*g* for 10 min and supernatants were stored at −20 °C until spectrophotometric and HPLC analyses.

### Spectrophotometric phytochemical analysis

Total phenols and total flavonoids were determined according to Zhishen *et al.* (*26*). For total phenol determination, 2 μL of test solution were diluted with 158 μL of deionized water and then 10 μL of Folin-Ciocalteu reagent were added. Afterwards, 30 μL Na_2_CO_3_ (1.88 M) were added, the mixture was incubated for 30 min at 45 °C and the absorbance was measured at 740 nm. Results were expressed as gallic acid equivalents (GAE). The content of total flavonoids was determined with AlCl_3._ First tested sample (20 μL) was diluted with 80 μL of dH_2_O, then a volume of 6 μL of 5% NaNO_2_ (*m*/*V*) was added. After 5 min of incubation, a volume of 6 μL of 10% AlCl_3_ (*m*/*V*) was added and the mixture was incubated at room temperature for additional 6 min. Afterwards, 40 μL NaOH (1 M) and distilled water were added to a final volume of 200 μL and absorbance was measured at 405 nm. Results were expressed as quercetin equivalents (QE). Total phenolic acids were determined according to European Pharmacopoeia (*27*). A volume of 40 μL of test solution was mixed with 80 μL HCl (0.5 M) and 80 μL of freshly prepared reagent (1 g NaNO_2_ and 1.17 g Na_2_MoO_4_·2H_2_O in 10 mL deionized water). The absorbance was measured at 492 nm and expressed as caffeic acid equivalents (CAE). Oxalic acid content was determined with KMnO_4_ according to the method described by Naik *et al.* (*28*). A volume of 25 μL of test solution was mixed with 125 μL of 1 M H_2_SO_4_ and 50 μL of 0.003 M KMnO_4_, incubated for 10 min, then absorbance was measured at 520 nm and the results expressed as oxalic acid equivalents (OAE). The nitrate content was determined using salicylic acid according to Cataldo *et al.* (*29*). A volume of 50 μL of test solution was mixed with 160 μL of 5% salicylic acid solution (*m*/*V*) in H_2_SO_4_. After 20 min of incubation at room temperature, 950 μL of 2 M NaOH were added, absorbance was measured at 405 nm and the results were expressed as KNO_3_ equivalents.

### Reversed-phase high-performance liquid chromatography analysis

Before HPLC analysis, samples were hydrolyzed with 1.2 M HCl for 2 h at 80 °C and 300 rpm. Qualitative and quantitative RP-HPLC analysis was performed using the Agilent 1100 Series system equipped with a quaternary pump, multiwave UV/Vis detector, autosampler, fraction collector, Zorbax SB C-18 analytical guard column (12.5 mm×4.6 mm, 5 µm particle size) and Poroshell 120 SB-C18 column (75 mm×4.6 mm, 2.7 µm particle size; Agilent Technologies, Waldbronn, Germany). The solvents used were: 0.2% glacial acetic acid (A), and 80% methanol and 0.2% glacial acetic acid (B). Gradient profile was (A/B): 100/0 at 0 min, 20/80 at 42 min, 0/100 at 43 min, 0/100 at 45 min, 100/0 at 45.1 min, 100/0 at 48 min as used by Šola *et al*. (*30*). Injection volume was 25 µL, the constant flow rate 1.0 mL/min, and the column temperature was set at 30 °C. For quantification, the multiwave UV/Vis detector was set at 254 nm for l-ascorbic acid, 310 nm for *p*-coumaric and ferulic acids, and 360 nm for quercetin determination. Phenolic compounds and l-ascorbic acid were characterized according to their retention times and UV spectra compared with commercial standards. For the quantitative analyses, calibration curves were obtained by injection of five known concentrations (in the range 1−250 µg/mL) of the mixed standard solution in triplicate. The results were expressed on dry mass basis in mg/kg.

### Antioxidant activity assays

DPPH radical scavenging and ferric reducing antioxidant power (FRAP) assay were performed as reported by Šola *et al.* (*30*) adapted to small volumes. The results of DPPH method are expressed in percentage of inhibition of DPPH and in μM of Trolox equivalents (TE) and of FRAP method in percentage of reduction of Fe^3+^-TPTZ complex and in μM Fe^2+^. Briefly, for DPPH method, 10 μL of test solution were added to 190 μL of freshly prepared ethanolic DPPH solution (0.1 mM) and incubated in the dark for 30 min at room temperature. The decrease in absorbance was measured at 520 nm. A calibration curve was constructed for Trolox and expressed as Trolox equivalents (TE). DPPH radical inhibition (%) was calculated as follows:

DPPH inhibition=((*A*_control_–*A*_sample_)/*A*_control_)·100 /1/

where *A*_control_ was the absorbance of the control (blank, without test solution) and *A*_sample_ was the absorbance in the presence of the test solution. 

For FRAP method, test solution (10 μL) was mixed with 190 μL of freshly prepared FRAP reagent (25 mL of 0.3 Μ CH_3_COONa·3H_2_O, pH=3.6, 2.5 mL of 10 mM TPTZ solution in 40 mM HCl and 2.5 mL of 20 mM FeCl_3_·6H_2_O) and the absorbance was read at 595 nm after 4 min of reaction time. A calibration curve was constructed for FeSO_4_·7H_2_O and the results were expressed in μM Fe^2+^ and in percentage of Fe^3+^-TPTZ complex reduction. FRAP reduction (%) was calculated as follows:

FRAP reduction=((*A*_sample_–*A*_control_)/*A*_sample_)·100 /2/

where *A*_control_ was the absorbance of the control (blank, without test solution) and *A*_sample_ was the absorbance in the presence of the test solution.

Trolox was used as a positive control for both methods when the results were presented in percentage.

### Assay of α-amylase inhibitory activity

Antidiabetic properties of predigested spinach lemon juice formulations and lemon juice through inhibition of α-amylase were tested using the preincubation method as described by Šola *et al.* (*25*) and expressed in percentage of inhibition. Predigested spinach lemon juice formulations and lemon juice (20 μL) were mixed with 20 μL α-amylase from human saliva (5 U/mL solution in ice-cold distilled water) and 40 μL of 20 mM phosphate-buffered saline (pH=6.9) and preincubated for 15 min at 37 °C. A volume of 20 μL of potato starch (1% *m*/*V* in 20 mM phosphate-buffered saline, pH=6.9) was added after the preincubation. The final concentration of predigested spinach lemon juice formulations was 6 mg/mL and of α-amylase it was 1 U/mL. Lemon juice was also diluted to a final volume fraction of 4%. After 15 min of incubation at 37 °C, 50 μL of dinitrosalicylic acid reagent (12 mL of distilled water, 8 mL of 5.3 M potassium sodium tartrate tetrahydrate solution in 2 M NaOH and 20 mL of 96 mM 3,5-dinitrosalicylic acid solution) were added and incubated at 85 °C for 15 min. A volume of 450 μL of distilled water was mixed with test solution and the absorbance was measured at 545 nm. Appropriate blanks and controls were prepared. The α-amylase enzyme inhibitory activity (%) was calculated as follows:

Amylase inhibition=100-((*A*_sample_–*A*_sample blank_)/(*A*_control_–*A*_control blank_) ·100) /3/

where *A*_sample_ was the absorbance of the test solution (with amylase), *A*_sample blank_ was the absorbance of the blank test solution (without amylase), *A*_control_ was the absorbance of the control (with amylase) and *A*_control blank_ was the absorbance of the blank control (without amylase).

Maltose was used as a positive control. All absorbance measurements were performed with microplate reader Fluostar Optima (BMG Labtech GmbH, Offenburg, Germany).

Bioaccessibility of phytochemicals

The bioaccessibility (%) was calculated with the following equations:

Bioaccessibility in salivary phase=(BA_salivary phase_/BA_initial phase_)·100 /4/

Bioaccessibility in gastric phase=(BA_gastric phase_/BA_initial phase_)·100 /5/

Bioaccessibility in intestinal phase=(BA_intestinal phase_/BA_initial phase_)·100 /6/

where BA_salivary phase_, BA_gastric phase_ and BA_intestinal phase_ corresponded to the total or individual bioactive compound concentration in salivary, gastric and intestinal phase and BA_initial_ was the total or individual bioactive compound concentration in the initial phase.

### Statistical analysis

All results were evaluated using STATISTICA v. 14.0.0.15 software (*31*). Results of RP-HPLC and spectrophotometric determination were subjected to one-way ANOVA for comparison of mean values, and significant differences were calculated according to Duncan's multiple range test. The data are presented as mean value±standard deviation (S.D.). Pearson’s correlation coefficient and principle component analysis (PCA) of phytochemicals, antioxidant and antidiabetic activity was performed. Data were considered statistically significant at p≤0.05.

## RESULTS AND DISCUSSION

### Phytochemical analysis

Total phenolic, total flavonoid, total phenolic acid, oxalic acid and nitrate contents of predigested and digested spinach lemon juice formulations are presented in Table 1. Significant increases in total phenolic mass fraction were noted after the addition of 20% lemon juice in predigested and almost all digested spinach samples with the exception of total phenols in the initial phase of digestion. During that phase significant increases were noted in spinach samples with 10% lemon juice in comparison with spinach formulated with 0% lemon juice. Total flavonoid and total phenolic acid mass fractions were significantly higher in all predigested spinach samples with lemon juice than in the predigested spinach sample without lemon juice. The highest total phenolic mass fraction was detected in predigested spinach samples with 20% lemon juice and in salivary and gastric phases, while the highest total phenolic acid mass fraction was detected in predigested samples and at all phases of digestion. Significant increase of the amount of total polyphenolic groups in predigested spinach lemon juice formulation can be due to lemon juice acidity which affected the stability of the tested total polyphenolic groups in acidic solutions (*17*). This phenomenon can best be seen in the spinach lemon formulation with the highest volume fraction of lemon juice. The ranges of total phenolic, total flavonoid and total phenolic acid contents on dry mass basis were 8.5-15.7, 6.3-9.2 and 1.8-4.5 mg/g, respectively. The results for total phenols were within the range reported by Turkmen *et al.* (*32*) and higher than those reported by Bunea *et al.* (*33*) and Roberts and Moreau *et al.* (*2*) for fresh, refrigerated processed spinach or dried spinach extract. Roberts and Moreau (*2*) also reported lower values of total flavonoids and total phenolic acids in fresh spinach. Oxalic acid mass fraction was statistically higher in spinach lemon juice formulations (*φ*(lemon juice)=2, 10 and 20% in original samples; 5, 10 and 20% in initial phase and 2, 5, 10 and 20% in salivary, gastric and intestinal phases) in a dose-dependent manner than in spinach samples. The range of oxalic acid mass fraction was 6.0−12.2 mg/g. Similar values were recorded by Akhtar *et al.* (*34*) and Savage *et al.* (*35*) in raw or boiled spinach and they were ten times lower than those found by Albert *et al.* (*36*) in dried spinach water extract. Average oxalate dry mass fraction in this study was 8.97 mg/g whereas Wang *et al.* (*4*) observed higher average oxalate mass fraction on fresh mass basis of 20.76 mg/g. According to Akhtar *et al.* (*34*) raw spinach contains about 55% soluble and 45% insoluble (mostly calcium) oxalates and both can be significantly reduced after boiling. Boiling can result in 50% of oxalic acid reduction (*5*). Insoluble oxalates were removed from our spinach water extract by filtration. Boiling and filtration may be the reason why we obtained lower oxalic acid values in spinach lemon juice formulations. Significantly higher nitrate mass fraction was noted in predigested spinach samples with the addition of 5, 10 and 20% lemon juice and in digested spinach sample with the addition of 20% lemon juice (salivary and intestinal phases). Nitrate mass fraction was in range of 7.4−20.5 mg/g. Average nitrate mass fraction in our study was 14.1 mg/g. Ten times lower average values of dietary nitrate were observed by Wang *et al.* (*4*) in fresh spinach. Morgado *et al.* (*7*) reported ten times lower average values of total phenols and 1.6 and 2.3 times lower nitrate values in nitrate-rich beetroot gel and juice before and after *in vitro* digestion, respectively. Higher values of total phenols and nitrates before and after *in vitro* digestion in our study were most likely obtained due to the usage of fine spinach powder, which is supported by the results found in Prasedya *et al.* (*24*) study. According to that group of authors, smaller particle size has a positive impact on the extraction of bioactive compounds. When we calculate the mean values of total phenols in all spinach lemon juice treatments, we can see their highest mass fraction in the oral phase of *in vitro* digestion, an insignificant decrease in the gastric phase, significant decrease in the initial and then in the intestinal phase, and the lowest values in the original samples. Spinach formulated with 20% of lemon juice had the highest total phenol mass fractions. Therefore, the addition of 20% lemon juice can increase the stability of spinach total phenols. The highest mean values of total flavonoid mass fraction in all treatments were observed before digestion (original samples) and in the initial and salivary phases of digestion, and significant drop was observed in the gastric and intestinal phases. The same trend was observed in predigested and digested spinach samples formulated with 20% lemon juice. In other formulations the stated stability of total flavonoids was not observed. Mean values for flavonoids and oxalic acid decreased in a series from the initial to the intestinal phase. The highest mean values of total phenolic acids in all treatments were observed in the initial and salivary phases, then a significant drop was observed in the intestinal, then in gastric phase and the lowest values were detected before *in vitro* digestion in original samples. This trend was observed in almost all individual spinach lemon juice formulations. A significant gradual decline in the mean values of nitrate mass fraction from the initial to the intestinal phase in all treatments was visible and the lowest values of nitrate were reported in original samples. Wootton-Beard *et al.* (*37*) detected increase in total phenolic content after gastric phase in all 23 tested vegetable juices, some of which contained lemon juice and spinach, and decrease or increase after the intestinal phase. Morgado *et al*. (*7*) detected an increase in total phenolic content after gastric and duodenal phases, and an increase in nitrate content after gastric and decrease after the intestinal phase of *in vitro* digestion. Significant increase of total phenolic content in matcha tea was observed in the gastric phase of *in vitro* digestion and then a decrease in the intestinal phase (*17*). The highest values of total flavonoids in matcha tea were observed in the initial and salivary phases, and a significant drop was observed in the gastric and then intestinal phase (*17*). The content of total phenolics, total flavonoids, total phenolic acids and nitrate in pure lemon juice was 3, 2, 3.5 and 13 times, respectively, smaller than in the predigested spinach sample, while lemon juice is 1.3 times richer source of oxalate than predigested spinach samples (Table 2). According to the data, the addition of lemon juice does not contribute to the contents of total phenolics, total flavonoids, total phenolic acids and nitrates in spinach lemon juice formulation, but it contributes to the content of the oxalic acid in dose-dependent manner. Also, lemon juice can accelerate polyphenol extraction due to the change in the pH of the extraction solvent (*38*). We reported significant decrease of pH in dose-dependent manner in predigested lemon juice formulation (0, 2, 5, 10 and 20%) (Table S1). This pH trend was also observed for initial, salivary, gastric and intestinal phases of digestion. The pH of pure lemon juice was 2.35 (Table 2) and it was statistically lower (2.2 times) than of predigested spinach sample. According to the results, pH of lemon juice can positively influence the number of polyphenolic groups.

**Table 1:** The total polyphenols, total flavonoids, total phenolic acids, oxalic acid and nitrate mass fractions on dry mass basis in predigested (original sample) and digested (initial phase, salivary phase, gastric phase and intestinal phase) spinach (S) and lemon juice (L) formulations

**Sample**	**Original sample**	**Initial phase**	**Salivary phase**	**Gastric phase**	**Intestinal phase**
***w*(total phenols as GAE)/(mg/g)**					
**S**	(8.6±0.3)^bC^	(10.4±0.7)^bABC^	(11.6±0.6)^bA^	(10.9±2.1)^bcAB^	(9.6±0.8)^bBC^
**S+L_2_**	(8.5±0.5)^bC^	(10.8±0.8)^abAB^	(12.2±1.6)^bA^	(9.6±1.2)^cBC^	(9.3±1.1)^bBC^
**S+L_5_**	(8.8±0.6)^abC^	(11.0±1.0)^abAB^	(12.3±1.2)^bA^	(11.8±1.4)^bcA^	(9.9±0.8)^abBC^
**S+L_10_**	(8.8±0.7)^abB^	(12.4±1.1)^aA^	(13.3±0.8)^abA^	(13.6±2.9)^abA^	(9.4±2.1)^bB^
**S+L_20_**	(9.6±0.7)^aC^	(11.3±1.7)^abB^	(14.9±1.6)^aA^	(15.7±0.8)^aA^	(11.9±1.4)^aB^
**Mean**	(8.9±0.7)^D^	(11.3±1.2)^B^	(12.9±1.6)^A^	(12.2±2.7)^AB^	(10.0±1.6)^C^
***w*(total flavonoids as QE)/(mg/g)**					
**S**	(6.3±2.3)^bB^	(8.8±0.1)^aA^	(7.96±0.09)^bAB^	(6.8±0.1)^bcB^	(7.5±0.3)^aAB^
**S+L_2_**	(8.85±0.09)^aA^	(7.9±0.1)^bB^	(8.02±0.03)^bB^	(6.7±0.1)^cC^	(6.76±0.07)^bcC^
**S+L_5_**	(9.1±0.3)^aA^	(7.7±0.1)^bB^	(7.8±0.4)^bB^	(6.97±0.09)^bcC^	(6.5±0.1)^cC^
**S+L_10_**	(9.1±0.2)^aA^	(8.3±0.7)^aA^	(8.07±0.05)^bB^	(7.1±0.4)^bC^	(6.7±0.4)^bcC^
**S+L_20_**	(8.92±0.08)^aA^	(8.6±0.4)^aA^	(9.2±1.1)^aA^	(7.63±0.08)^aB^	(7.1±0.4)^abB^
**Mean**	(8.5±1.4)^A^	(8.4±0.6)^A^	(8.2±0.7)^A^	(7.0±0.4)^B^	(6.9±0.4)^B^
***w*(total phenolic acids as CAE)/(mg/g)**					
**S**	(1.8±0.1)^dE^	(3.55±0.06)^dB^	(3.8±0.1)^dA^	(2.9±0.1)^cD^	(3.2±0.2)^cC^
**S+L_2_**	(2.20±0.06)^bD^	(4.1±0.3)^bcA^	(3.9±0.1)^cdA^	(3.0±0.2)^cC^	(3.5±0.2)^cB^
**S+L_5_**	(2.09±0.03)^cD^	(3.9±0.1)^cA^	(4.0±0.1)^bcA^	(3.1±0.1)^bcC^	(3.44±0.08)^cB^
**S+L_10_**	(2.12±0.03)^bcD^	(4.3±0.3)^abA^	(4.2±0.1)^bA^	(3.3±0.3)^bC^	(3.8±0.2)^bB^
**S+L_20_**	(2.30±0.04)^aC^	(4.5±0.3)^aA^	(4.5±0.2)^aA^	(3.8±0.2)^aB^	(4.4±0.2)^aA^
**Mean**	(2.1±0.2)^D^	(4.1±0.4)^A^	(4.1±0.3)^A^	(3.2±0.4)^C^	(3.7±0.4)^B^
***w*(oxalic acid as OAE)/(mg/g)**					
**S**	(7.5±1.00)^dB^	(9.30±0.01)^dA^	(9.22±0.06)^eA^	(7.98±0.01)^eB^	(6.02±0.07)^eC^
**S+L_2_**	(9.2±0.2)^cB^	(9.47±0.01)^cdA^	(9.4±0.1)^dAB^	(8.11±0.03)^dC^	(6.31±0.04)^dC^
**S+L_5_**	(8.4±1.1)^cdB^	(9.5±0.3)^cA^	(9.63±0.03)^cA^	(8.34±0.01)^cB^	(6.53±0.02)^cC^
**S L_10_**	(10.4±0.6)^bA^	(10.23±0.01)^bA^	(10.19±0.05)^bA^	(8.80±0.01)^bB^	(6.90±0.06)^bC^
**S+L_20_**	(12.24±0.01)^aA^	(11.54±0.02)^aB^	(11.53±0.01)^aB^	(9.91±0.01)^aC^	(7.71±0.06)^aD^
**Mean**	(9.5±1.8)^A^	(10.0±0.9)^A^	(10.0±0.9)^A^	(8.6±0.7)^B^	(6.7±0.6)^B^
***w*(nitrate as KNO_3_E)/(mg/g)**					
**S**	(7.4±0.2)^cE^	(19.67±0.09)^abA^	(16.1±1.1)^bB^	(13.1±0.3)^cC^	(10.2±0.5)^bD^
**S+L_2_**	(8.2±0.2)^bcE^	(19.3±0.3)^bA^	(16.7±0.9)^abB^	(13.5±0.5)^bcC^	(10.0±0.4)^bD^
**S+L_5_**	(9.21±0.06)^abD^	(19.5±0.3)^bA^	(17.2±1.2)^abB^	(13.5±0.2)^abcC^	(10.6±0.3)^bD^
**S+L_10_**	(9.9±1.2)^aC^	(20.2±0.3)^abA^	(16.8±1.3)^abB^	(14.4±0.2)^aB^	(10.5±1.1)^bC^
**S+L_20_**	(10.4±0.2)^aE^	(20.5±0.5)^aA^	(19.0±0.4)^aB^	(14.4±0.5)^abC^	(12.3±0.3)^aD^
**Mean**	(9.0±1.2)^E^	(19.8±0.5)^A^	(17.2±1.3)^B^	(13.8±0.618)^C^	(10.7±1.0)^D^

Data are presented as mean value±S.D, *N*=4. Different lower case letters indicate significant difference at p≤0.05 within each phase separately. Capital letters indicate significant difference at p≤0.05 among phases. *φ*(lemon juice)=2 (L_2_), 5 (L_5_), 10 (L_10_) and 20 (L_20_) %. GAE=gallic acid equivalents, QE=quercetin equivalent, CAE=caffeic acid equivalents, OAE=oxalic acid equivalents, KNO_3_E=KNO_3_ equivalents

**Table 2:** Phytochemical content on dry mass basis, antioxidant activity, antidiabetic activity and pH of pure lemon juice

*w*(TP as GAE)(mg/g)	*w*(OAE)/(mg/g)	*w*(*p*-CA)/(mg/kg)	DPPH inhibition/%	FRAP reduction/%
2.91±0.12	9.82±0.02	5.87±0.09	80.2±1.8	93.4±0.3
*w*(TF as QE)/(mg/g)	*w*(nitrate as KNO_3_E)/(mg/g)	*w*(FA)/(mg/kg)	DPPH as *b*(TE)/(μmol/g)	FRAP as *b*(Fe^2+^E)/(μmol/g)
3.22±0.11	0.56±0.05	16.2±0.6	2642±60	3251±180
*w*(TPA as CAE)/(mg/g)	*w*(l-AA)/(mg/kg)	*w*(Q)/(mg/kg)	α-amylase inhibition/%	pH
0.52±0.02	1520±112	30.5±0.7	81.9±6.0	2.35±0.02

Data are presented as mean value±S.D, *N*=4. TP=total polyphenols, GAE=gallic acid equivalents, TF=total flavonoids, QE=quercetin equivalents, TPA=total phenolic acids, CAE=caffeic acid equivalents, OAE=oxalic acid equivalents, KNO_3_E=KNO_3_ equivalents, l-AA=l-ascorbic acid, *p*-CA=*p*-coumaric acid, FA=ferulic acid, Q=quercetin, TE=Trolox equivalents, Fe^2+^E= Fe^2+^ equivalents

**Fig. 1:**
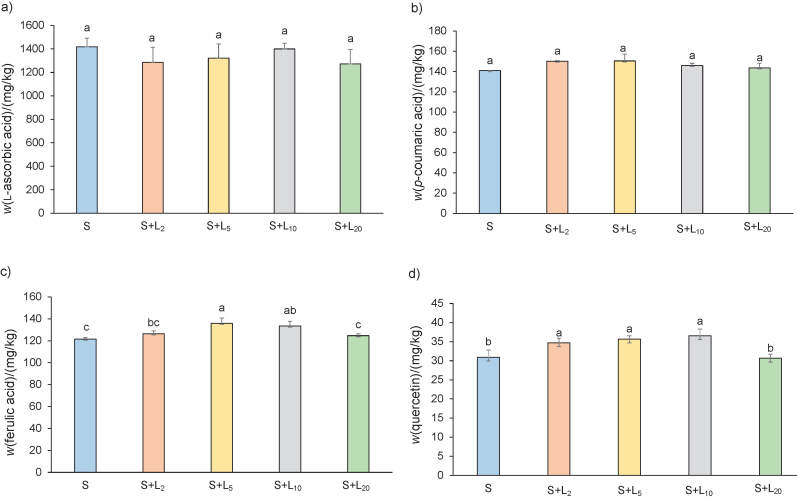
RP-HPLC analysis of: a) l-ascorbic acid and individual phenolic compounds on dry mass basis, b) *p*-coumaric acid, c) ferulic acid and d) quercetin in predigested (original sample) formulations of spinach (S) with *φ*(lemon juice)=2, 5, 10 and 20% (L_2_, L_5_, L_10_ and L_20_). Data are presented as mean value±S.D, *N*=3. Different letters indicate significant differences at p≤0.05

**Fig. 2:**
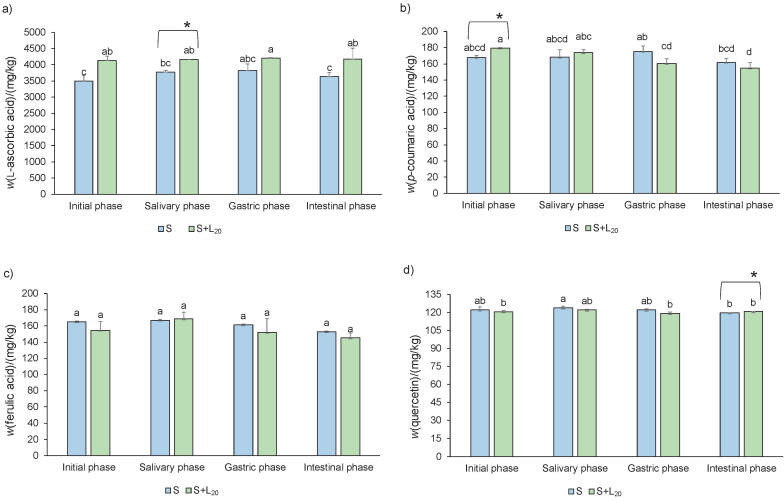
RP-HPLC analysis of: a) l-ascorbic acid and individual phenolic compounds on dry mass basis, b) *p*-coumaric acid, c) ferulic acid and d) quercetin in digested (initial phase, salivary phase, gastric phase and intestinal phase) spinach (S) and *φ*(lemon juice)=20% (L_20_) formulations. Data are presented as mean value±S.D, *N*=3. Different letters indicate significant differences at p≤0.05 for all samples and phases together. Asterisk indicates significant differences at p≤0.05 for each phase individually

The mass fraction of l-ascorbic acid and individual phenolic compounds (*p*-coumaric acid, ferulic acid and quercetin) in predigested spinach lemon juice formulations is shown in Fig. 1. There was no significant increase in l-ascorbic acid (Fig. 1a) and *p*-coumaric acid (Fig. 1b) in all the tested predigested spinach lemon juice formulations. The highest mass fraction of ferulic acid (Fig. 1c) was observed in predigested spinach samples with the addition of 5% lemon juice and the lowest mass fraction was observed in the spinach formulations with 0 and 20% of lemon juice. Quercetin mass fraction (Fig. 1d) was significantly higher in spinach formulations with 2, 5 and 10% lemon juice than in spinach formulation with 0 and 20% lemon juice. Mass fractions of l-ascorbic acid, *p*-coumaric acid, ferulic acid and quercetin in lemon juice are shown in Table 2. Lemon juice is richer source of l-ascorbic acid by 1.1 time than predigested spinach samples. The mass fraction of *p*-coumaric acid, ferulic acid and quercetin was 24.0, 7.5 and 1.0 times, respectively, lower than in the predigested spinach sample. Accordingly, the addition of lemon juice does not contribute to the contents of individual phenolic compounds, but it contributes to the content of l-ascorbic acid in spinach lemon juice formulation. l-ascorbic acid and individual phenolic compounds were analysed in digested samples with 0 and 20% lemon juice by (Fig. 2). Significant increase in the mass fraction of l-ascorbic acid (Fig. 2a) was noted in the salivary phase in spinach formulated with 20% lemon juice when each phase was analyzed individually. When data of all samples and phases were analyzed together, a significant increase of l-ascorbic acid was detected in the initial and intestinal phases of digestion. The addition of lemon juice to spinach increased l-ascorbic acid mass fraction in the initial, salivary, gastric and intestinal phases of digestion for 1.2, 1.1, 1.1 and 1.2 times, respectively, compared to the pure spinach formulation. Mass fraction of  *p*-coumaric acid (Fig. 2b) significantly increased in the initial phase and of quercetin (Fig. 2d) in the intestinal phase of digestion in spinach formulated with 20% lemon juice when each phase was analyzed individually. When data of all samples and phases were analyzed together, significant decrease of *p-*coumaric acid (Fig. 2b) was observed in the gastric phase of digestion in spinach formulated with 20% lemon juice. There was no significant increase in the ferulic acid (Fig. 2c) mass fraction in all phases of *in vitro* digestion between spinach formulations with 0 and 20% lemon juice. In predigested and digested spinach samples, the mass fraction of l-ascorbic acid, *p*-coumaric acid, ferulic acid and quercetin on dry mass basis was in the range of 1273−4200, 140.9−179.4, 121.6−168.4 and 30.9−124.0 mg/kg, respectively. In the study of Delchier *et al.* (*39*), mass fraction of vitamin C in spinach samples varied from 74 to 170 mg/kg after boiling. These results were about twenty times lower than in our study. Bunea *et al.* (*33*) detected lower mass fraction on fresh mass of *o*-coumaric acid (28–60 mg/kg), ferulic acid (10–35 mg/kg) and *p*-coumaric acid (1–30 mg/kg) in fresh, refrigerated and processed spinach. They reported an increase in the mass fraction of all phenolic acids after boiling due to the degradation of complex phenolic structures (tannins or flavonoids) into simple phenolics (phenolic acids). According to Roberts and Moreau (*2*), spinach contains negligible amounts of quercetin and kaempferol. High mass fractions of  l-ascorbic acid, nitrate, total and individual phenolic compounds in our study compared to the results presented by other authors can be due to the usage of fine dry spinach powder for sample preparation. We prepared the samples by cooking (40 min), during which the high temperature can destroy the cell walls of the dry spinach powder and release phytochemicals from the food matrix.

### Phytochemical bioaccessibility

Bioaccessibility of total and individual polyphenols, l-ascorbic acid, oxalic acid and nitrates is shown in Table 3. The mean values of oxalic acid and nitrate bioaccessibility percentage show their decline during *in vitro* digestion. The highest mean values of total phenolic and quercetin bioaccessibility were obtained in salivary phase and a significant drop was observed in the gastric and intestinal phase of *in vitro* digestion. No significant change was observed for l-ascorbic acid and *p*-coumaric acid bioaccessibility mean values. Significant drop in total phenolic and ferulic acid bioaccessibility mean values was observed in the intestinal phase, as opposed to the salivary phase. The highest mean value of total phenolic acid bioaccessibility was measured in salivary phase and the lowest in gastric phase of digestion. High bioaccessibility of oxalic acid in intestinal phase can be due to the degradation of complex polyphenols to simple phenolic acids after hydrolysis in gastric phase (acid environment, pH=2) of digestion. Bioaccessibility of all parameters was very high and in the range of 75.7−139.5% of total phenolics, 75.9−106.2% of total flavonoids, 73.4−105.9% of total phenolic acids, 86.2−104.6% of *p*-coumaric acid, 92.5−108.8% of ferulic acid, 98.1−101.5% of quercetin, 100.8−109.0% of l-ascorbic acid, 64.7−101.0% of oxalic acid and 51.5−92.9% of nitrates. The highest bioaccessibility of total phenolics (132.1% in salivary phase, 139.5% in gastric phase and 105.8% in intestinal phase) was observed in the spinach formulated with the highest volume fraction of lemon juice. In spinach samples formulated with 20% lemon juice, the highest percentage of bioaccessibility of total phenolic acids was in gastric (85.1) and intestinal (97.4) phases and of nitrate (60.2) and quercetin (100.1) in the intestinal phase of digestion. The highest bioaccessibility of oxalic acid (101.0% in salivary phase, 87.4% in gastric phase and 68.5% in intestinal phase) was observed in the spinach formulated with 5% lemon juice. Significantly higher l-ascorbic acid bioaccessibility (107.7%) in salivary phase was observed in the spinach without lemon juice than in the spinach formulated with the highest volume fraction of lemon juice. Wootton-Beard *et al*. (*37*) also detected high bioaccessibility of total phenolics in some vegetable juices which contained lemon juice and spinach. Green *et al.* (*14*) detected a significant increase in total catechin recovery in green tea formulated with 20 and 50% lemon juice. Digestive recovery of catechins from green tea was achieved by ascorbic acid from lemon juice (*14*). Bravo (*40*) reported that quercetin and free simple phenolic acids are directly absorbed through the small intestinal mucosa and Iqbal *et al.* (*41*) reported that l-ascorbic acid is absorbed in the buccal mucosa, stomach and the small intestine. Accordingly, all spinach lemon juice formulations represent a good source of high intestinal bioaccessibility of quercetin and individual phenolic acids, and high salivary, gastric and intestinal bioaccessibility of l-ascorbic acid. Morgado *et al.* (*7*) also detected a high percentage of nitrate bioaccessibility in nitrate-rich beetroot gel and juice. Nitrate from green leafy vegetables or beetroot is mainly reabsorbed in the mouth (in the salivary glands) and then in the proximal intestines (*8*). Bioaccessibility of nitrates from spinach lemon juice formulation was very high (81.7−92.9%) in the salivary phase in all samples and the highest (60.2%) in the intestinal phase in the spinach formulated with the highest lemon volume fraction (20%). Therefore, spinach formulated with 20% of lemon juice represents the best source of dietary nitrates. Savage and Catherwood (*42*) reported that oxalates are absorbed by small intestine or in the ileum. Bioaccessibility of oxalates from spinach lemon juice formulation was in the range of 64.7-68.5% in the intestinal phase of *in vitro* digestion. This percentage of oxalate bioaccessibility can be drastically reduced in human intestine depending on the coingestion of calcium, magnesium and fibre.

**Table 3:** Bioaccessibility of total and individual polyphenols, oxalic acid, nitrate and l-ascorbic acid in spinach lemon juice formulations

**Sample**	**Bioaccessibility/%**						
	Total phenolics						
	Salivary phase	Gastric phase	Intestinal phase				
**S**	(111.6±8.2)^abA^	(105.2±20.6)^abA^	(91.9±7.6)^abA^				
**S+L_2_**	(112.8±14.9)^abA^	(90.4±11.2)^bB^	(86.5±10.0)^abB^				
**S+L_5_**	(111.2±10.9)^abA^	(106.9±13.0)^abAB^	(90.2±7.7)^abB^				
**S+L_10_**	(107.3±6.4)^bA^	(109.5±23.7)^abA^	(75.7±16.9)^bB^				
**S+L_20_**	(132.1±13.3)^aA^	(139.5±6.8)^aA^	(105.9±11.8)^aB^				
**Mean**	(115.0±8.7)^A^	(110.3±16.0)^A^	(90.0±9.7)^B^				
		Total flavonoids		Total phenolic acids			
	Salivary phase	Gastric phase	Intestinal phase		Salivary phase	Gastric phase	Intestinal phase
**S**	(90.9±1.0)^bA^	(77.9±1.2)^cC^	(84.1±1.0)^aB^		(105.9±3.2)^aA^	(82.8±2.8)^abC^	(91.4±5.0)^abB^
**S+L_2_**	(101.6±0.4)^aA^	(84.6±1.7)^bB^	(85.7±1.0)^aB^		(93.7±2.4)^cA^	(73.4±5.6)^cC^	(84.0±4.5)^cB^
**S+L_5_**	(101.9±5.2)^aA^	(90.9±1.2)^aB^	(84.2±1.0)^aC^		(101.8±3.1)^abA^	(79.1±2,8)^abcC^	(88.0±1.9)^bcB^
**S+L_10_**	(91.4±0.6)^bA^	(80.4±4.6)^cB^	(75.9±1.0)^bB^		(97.2±3.2)^bcA^	(77.9±8.8)^bcC^	(88.3±4.9)^bcB^
**S+L_20_**	(106.2±12.3)^aA^	(88.2±0.9)^aB^	(81.6±1.0)^aB^		(100.9±4.6)^abA^	(85.1±3.8) ^aB^	(97.4±3.3)^aA^
**Mean**	(98.4±6.2)^A^	(84.4±4.8)^B^	(82.3±3.4)^B^		(100.0±4.1)^A^	(79.7±4.1)^C^	(89.8±4.5)^B^
		Oxalic acid		Nitrate			
	Salivary phase	Gastric phase	Intestinal phase		Salivary phase	Gastric phase	Intestinal phase
**S**	(99.1±0.7)^bcA^	(85.86±0.05)^bcB^	(64.7±0.7)^dC^		(81.7±5.4)^aA^	(66.5±1.7)^bB^	(51.9±2.8)^bC^
**S+L_2_**	(98.8±1.1)^cA^	(85.6±0.3)^cB^	(66.6±0.4)^cC^		(86.3±4.8)^aA^	(69.7±2.4)^abB^	(51.5±2.2)^bC^
**S+L_5_**	(101.0±0.3)^aA^	(87.4±0.2)^aB^	(68.5±0.3)^aC^		(88.0±6.0)^aA^	(69.2±0.9)^abB^	(54.4±1.6)^bC^
**S+L_10_**	(99.6±0.5)^bcA^	(85.9±0.1)^cB^	(67.5±0.6)^bC^		(83.3±6.5)^aA^	(71.5±0.8)^aA^	(52.1±5.3)^abB^
**S+L_20_**	(99.92±0.08)^bcA^	(85.87±0.07)^bcB^	(66.9±0.6)^bcC^		(92.9±1.9)^aA^	(70.1±1.7)^abB^	(60.2±1.2)^aC^
**Mean**	(99.7±0.8)^A^	(86.1±0.6)^B^	(66.8±1.2)^C^		(86.4±3.9)^A^	(69.4±1.6)^B^	(54.0±3.2)^C^
		l-ascorbic acid		*p*-coumaric acid			
	Salivary phase	Gastric phase	Intestinal phase		Salivary phase	Gastric phase	Intestinal phase
**S**	(107.7±1.7)^aA^	(109.0±6.0)^aA^	(103.8±3.5)^aA^		(100.3±5.5)^aA^	(104.6±4.1)^aA^	(96.5±2.9)^aA^
**S+L_20_**	(100.78±0.03)^bA^	(101.6±0.5)^aA^	(100.8±8.4)^aA^		(96.8±2.1)^aA^	(89.5±3.2)^aAB^	86.2±3.9)^aB^
**Mean**	(104.3±4.9)^A^	(105.3±5.2)^A^	(102.3±2.1)^A^		(98.5±2.5)^A^	(97.0±10.7)^A^	(91.3±7.1)^A^
		Ferulic acid				Quercetin	
	Salivary phase	Gastric phase	Intestinal phase		Salivary phase	Gastric phase	Intestinal phase
**S**	(101.2±4.1)^aA^	(97.7±5.7)^aA^	(92.5±0.4)^aA^		(101.5±1.0)^aA^	(99.9±0.8)^aAB^	(98.06±0.02)^bB^
**S+L_20_**	(108.8±5.5)^aA^	(98.2±11.0)^aA^	(93.8±3.9)^aA^		(101.2±0.5)^aA^	(98.8±0.9)^aAB^	(100.1±0.1)^aB^
**Mean**	(105.0±5.4)^A^	(98.0±0.4)^AB^	(93.2±0.9)^B^		(101.3±0.2)^A^	(99.3±0.7)^B^	(99.1±1.5)^B^

Data are presented as mean value±S.D, *N*=5 and *N*=2 for l-ascorbic acid, *p*-coumaric acid, ferulic acid and quercetin. Different lower case letters indicate significant differences at p≤0.05 within each phase. Capital letters indicate significant differences at p≤0.05 among all phases. S=spinach, *φ*(lemon juice)=2, 5, 10 and 20% (L_2_, L_5_, L_10_ and L_20_)

### Antioxidant activity

**Fig. 3:**
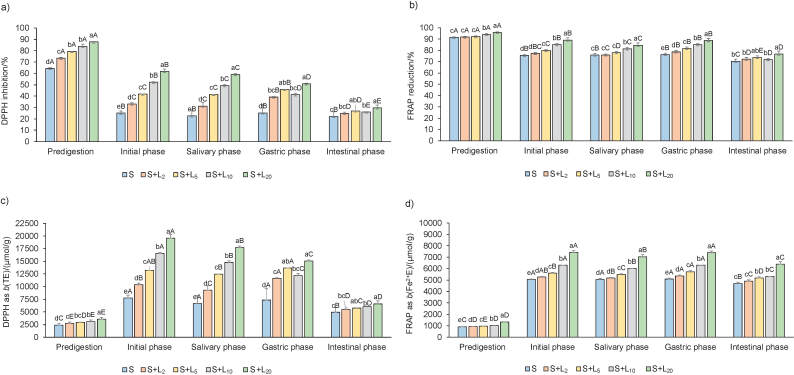
Antioxidant activity on dry mass basis: a) DPPH inhibition/%, b) FRAP reduction/%, c) DPPH as TE/(mµol/g), and d) FRAP as Fe^2+^E/(µmol/g) in predigested and digested (initial phase, salivary phase, gastric phase and intestinal phase) spinach lemon juice formulations. Data are presented as mean value±S.D, *N*=4. Different lower case letters indicate significant differences at p≤0.05 for each phase separately. Capital letters indicate significant differences at p≤0.05 between all phases together. S=spinach, *φ*(lemon juice)=2, 5, 10 and 20% (L_2_, L_5_, L_10_ and L_20_), DPPH=2,2-diphenyl-1-picrylhydrazyl, FRAP=ferric ion reducing antioxidant power

Antioxidant activity of predigested and digested spinach lemon juice formulations was estimated with DPPH and FRAP methods (Fig. 3). According to the DPPH method, when results are expressed in percentage (Fig. 3a), all predigested spinach lemon juice formulations and pure lemon juice (Table 2) samples have a strong antioxidant activity compared to Trolox (88%). Strong antioxidant activity, detected with DPPH method (Fig. 3a), was also noted in the spinach samples with 20% lemon juice in the initial and salivary phases. All predigested and digested spinach lemon juice formulations and pure lemon juice samples have a strong antioxidant activity, measured with FRAP method, expressed in percentage (Fig. 3b) compared to Trolox as a positive control (97%). Statistically the highest antioxidant activity expressed in percentage and in µmol/g (Figs. 3c and 3d) using both antioxidant methods was noted in predigested and all digested spinach samples with 20% lemon juice. In almost all predigested and digested spinach lemon juice formulations (*φ*=2, 5, 10 and 20%), there was a significant increase of antioxidant activity measured with both antioxidant methods, either expressed in percentage or in µmol/g in dose-dependent manner in comparison to plain spinach sample. Using FRAP method, we observed an increase in the antioxidant activity in the gastric phase and decrease after intestinal phase compared to salivary phase in almost all spinach lemon juice formulations (Figs. 3b and 3d), and in some spinach lemon juice formulations using DPPH method (Figs. 3a and 3c). This trend was also observed by Wootton-Beard *et al.* (*37*) using DPPH and FRAP methods, and in matcha tea by Rusak *et al.* (*17*) using ABTS, DPPH and FRAP methods. We assumed that an acid environment in gastric digestion (pH=2) positively affects polyphenols, which are known antioxidants. We confirmed this claim with Pearson correlation coefficients (Table S2). All total polyphenols (total phenolics, total flavonoids and total phenolic acids) and antioxidant activity parameters (expressed in percentage or as concentration) significantly correlated with each other in the gastric phase of digestion and had very strong (0.801-0.941) or strong correlations (0.634-0.795). This trend was observed in the initial and intestinal phases of digestion of total phenolics and total phenolic acids in both antioxidant activity methods. Very strong  (-0.822−0.964) or strong negative correlations (-0.773−0.788) in gastric phase between pH and total polyphenol, oxalic acid and nitrate contents and antioxidant activity were obtained (Table S2). Negative correlations between pH and the content of total phenolics (0.748), total phenolic acids (0.946), oxalic acid (0.966) and nitrates (0.714), and antioxidant activity measured by DPPH (0.976%, 0.973 µmol/g) and FRAP (0.932%, 0.980 µmol/g) assays were observed in the initial phase (Table S2). Negative correlation between pH and the content of total phenolic acids (0.714) and oxalic acid (0.747), and DPPH (0.902%, 0.904 µmol/g) and FRAP (0.756 µmol/g) antioxidant activity was observed in the intestinal phase (Table S2). This means that lowering the pH of spinach lemon juice formulation contributes to increasing the content of phytochemicals and antioxidant activity. Based on these correlations, we can conclude that oxalic acid affects pH. Oxalic acid is relatively strong acid which may function as a pH regulator in plants (*43*). Almost all antioxidant activity measurements correlated very strongly (>0.828-0.988) or strongly (0.718-0.769) with oxalic acid and strongly with nitrates in the initial, gastric and intestinal phases of digestion. According to the literature, oxalic acid can act as a natural antioxidant by reducing the rate of ascorbic acid oxidation in the presence of hydrogen peroxide and Cu^2+^ (*44*), and dietary nitrates can have positive effect on antioxidant capacity in animal model (*44*). According to the literature, both spinach and lemon juice are good sources of antioxidants (*2*, *11*, *14*, *32*, *45*). Antioxidant activity of pure lemon juice (Table 2) determined with both methods and expressed as percentage or concentration was 1.2, 1.1, 1.0 and 3.6 times higher than of the predigested spinach sample, respectively (Fig. 3).

### Inhibition of α-amylase

**Fig. 4:**
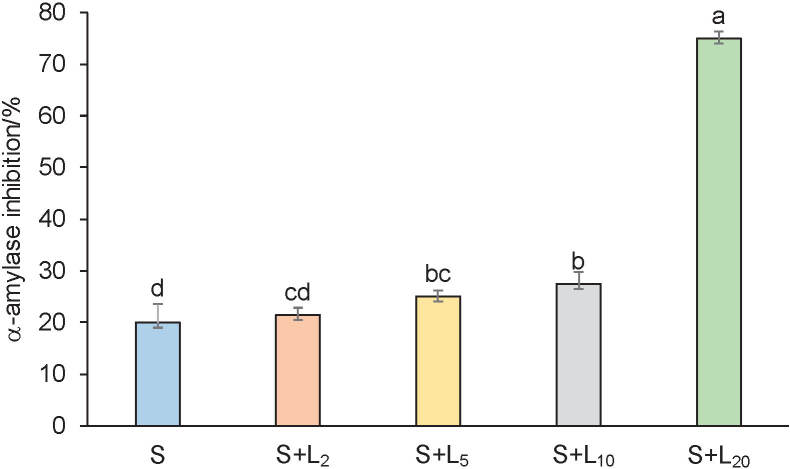
Activity of α-amylase inhibition of predigested spinach (S) with *φ*(lemon juice)=2, 5, 10 and 20% (L_2_, L_5_, L_10_ and L_20_) formulations. Data are presented as mean value±S.D, *N*=4. Different letters indicate significant differences at p≤0.05

Antidiabetic properties of predigested spinach lemon juice formulations and lemon juice measured by the inhibition of α-amylase are given in Fig. 4 and in Table 2. Strong α-amylase inhibitory activity was observed only in spinach samples with 20% lemon juice (75.0%) and in diluted lemon juice (81.9%) compared to maltose (90%) (Fig. 4). All other spinach lemon juice formulations had a weak α-amylase inhibitory activity of 20.0, 21.5, 25.1 and 27.5%, respectively, compared to maltose. Statistically significant increase of the percentage of inhibition of α-amylase was measured in 5, 10 and 20% spinach lemon juice formulation in dose-dependent manner in comparison to spinach sample. It seems that the addition of lemon juice contributes to α-amylase inhibitory activity of spinach in dose-dependent manner. α-Amylase inhibitory activity of pure lemon juice (Table 2) was 4 time higher than of the predigested spinach sample (Fig. 4). Gironés-Vilaplana *at al.* (*46*) reported that lemon and lime have good antidiabetic activity, which correlated with vitamin C and flavone contents. Rusak *et al.* (*17*) reported that antioxidant and antidiabetic activities depend on the concentration of total and individual polyphenolic compounds before and during *in vitro* digestion. In predigested samples (Table S3), α-amylase inhibitory activity correlated very strongly (>0.800) or strongly (>0.600<0.799) with antioxidant activity (FRAP: 0.859%, and 0.975 µmol/g, DPPH: 0.645% and 0.811 µmol/g), total phenolics (0.755), total phenolic acids (0.774), oxalic acid (0.797) and nitrate (0.646) contents. Khalifi *et al.* (*44*) reported positive outcomes of dietary nitrate on glucose tolerance and antioxidant capacity in type 2 diabetic rats. Very strong (-0.811−0.970) or strong negative correlations (-0.738−0.780) in predigested samples between pH and almost all total polyphenols, oxalic acid, nitrates and antioxidant activity, and moderate negative correlation (-0.651) between pH and α-amylase inhibitory activity were obtained (Table S3). Accordingly, low pH enhances the extraction of phytochemicals that have strong positive influence on antioxidant and moderate on antidiabetic activity.

### Principal component analysis

**Fig. 5:**
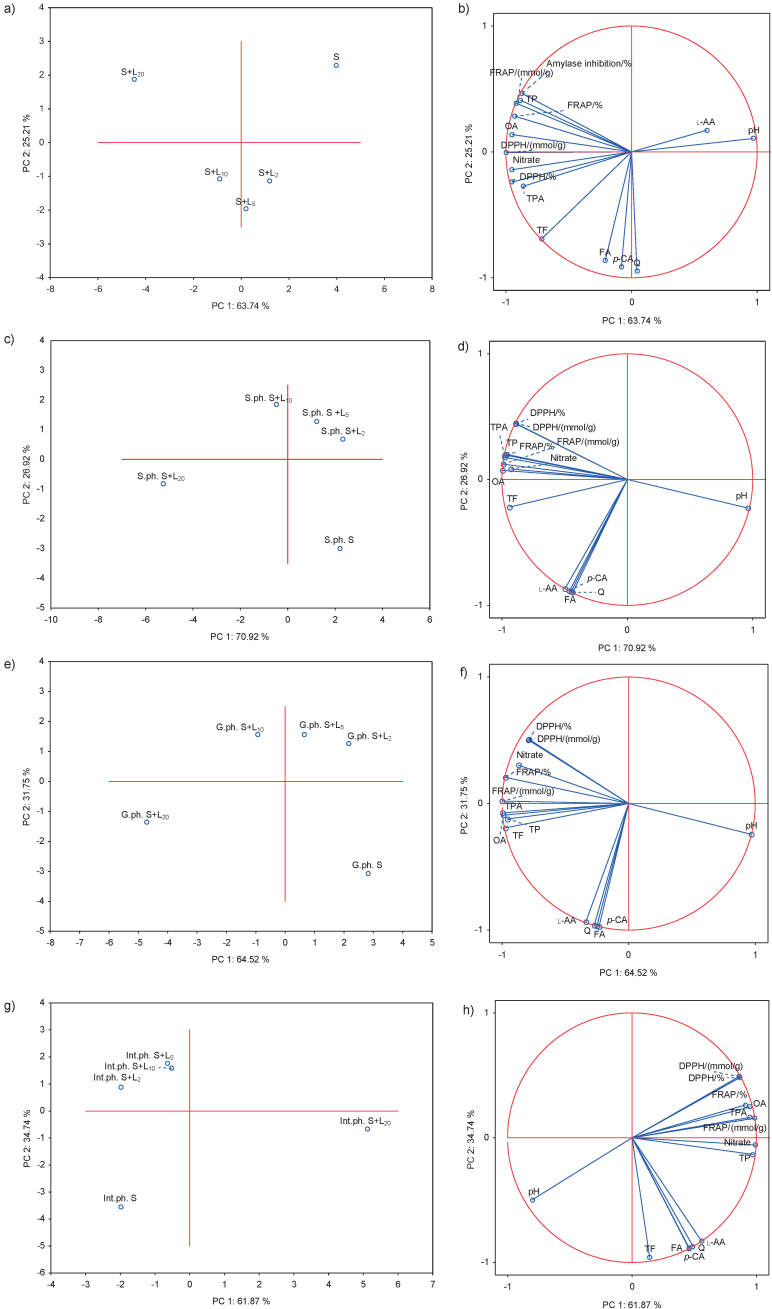
The principal component analysis (PCA) performed on the correlation matrix of average values of pH, TP=total phenols, TF=total flavonoids, TPA=total phenolic acids and individual phenolic compounds (*p*-CA=*p*-coumaric acid, FA=ferulic acid and Q=quercetin), l-AA=l-ascorbic acid, OA=oxalic acid, and nitrate, antioxidant activity (DPPH inhibition/% and as *b*(TE)/(μmol/g), FRAP/% and as *b*(Fe^2+^E)/(μmol/g) and antidiabetic (α-amylase inhibition/%) activity in predigested and digested, salivary phase (S.ph.), gastric phase (G.ph.) and intestinal phase (Int.ph.) of spinach (S) and *φ*(lemon juice)=2, 5, 10 and 20% (L_2_, L_5_, L_10_ and L_20_) formulations: a, c, e and g) score plot separating the spinach lemon formulation, and b, d, f and h) the loading plot of pH, metabolites and antioxidant and antidiabetic activity as variables

The PCA plots provide an overview of the similarities and differences among different spinach lemon juice formulations as well as the interrelationships between the measured properties (pH, phytochemical composition, antioxidant and antidiabetic activities) (Fig. 5). The first (PC 1) and the second principal component (PC 2) described 63.74 and 25.21%, respectively, of the variance for predigested samples (Fig. 5a), 70.92 and 26.92% of the variance for salivary (Fig. 5c), 64.52 and 31.75% of the variance for gastric (Fig. 5e) and 61.87 and 23.74% of the variance for intestinal phases (Fig. 5g). Together, the first two PCs represent 88.95% of the total variability for predigested (Fig. 5a), 97.84% for salivary (Fig. 5c), 96.27% for gastric phase (Fig. 5e) and 96.61% for intestinal phase (Fig. 5g). The greatest distance that points to the biggest difference was detected between the spinach without and with 20% lemon juice in predigested and in all digested samples (Fig. 5). The smallest distance which points to the smallest difference was detected between the spinach formulations with 2, 5 and 10% lemon juice in predigested (Fig. 5a) and in digested samples (Figs. 5c, 5e and 5g). Spinach formulated with 20% lemon juice had strong loadings with most tested total (total phenolic, total phenolic acid, oxalic acid and nitrates) compounds, and antioxidant and α-amylase inhibitory activities in predigested stage (Fig. 5b). According to the above mentioned methods, spinach formulated with the highest volume fraction of lemon juice had the highest mean values of all parameters. Spinach without lemon juice had the highest measured values of l-ascorbic acid and pH compared to other formulations. In predigested samples, the highest distance which points to the biggest difference was detected between l-ascorbic acid and pH and all other methods. These results indicate that the contents of total polyphenolic compounds, oxalates and nitrates in predigested samples are closely related to antioxidant and α-amylase inhibitory activities and that lowering the pH value with lemon juice can positively affect these parameters, which is confirmed by the literature (*17*, *38*, *43*, *44*). In all digested samples (Figs. 5c−5h) spinach formulated with the highest volume fraction of lemon juice had high content of almost all tested total polyphenolics, nitrates, oxalates and individual compounds measured by HPLC (l-ascorbic acid, *p*-coumaric acid, ferulic acid and quercetin), and strong antioxidant activity. Also, pure spinach formulation without lemon juice had higher pH value than all other spinach lemon formulation (2, 5, 10 and 20%). Accordingly, polyphenolic compounds and l-ascorbic acid in almost all digested samples are closely related with antioxidant activity, which is consistent with the literature (*25*). The highest distance was detected between pH and antioxidant activity in digested samples. This means that by reducing the pH by adding lemon juice in higher volume fractions contributes to the stabilization of polyphenols and vitamin C in *in vitro* digestion, which directly affects the increase of antioxidant activity of spinach formulation.

## CONCLUSIONS

This is the first report about the stability and digestive availability of spinach polyphenols, oxalic acid, nitrate and l-ascorbic acid in spinach lemon formulations. High bioavailability of almost all tested compounds can be attributed to the preparation of samples with fine powder and concentration of the sample by cooking for 40 min, during which the high temperature can destroy the cell walls of the dry spinach powder and release the phytochemicals from the food matrix. Lemon juice pH and antioxidants (l-ascorbic acid) increase the stability and availability of polyphenols in spinach lemon juice formulations during *in vitro* digestion and positively influence antioxidant activity. Addition of lemon juice to spinach contributes to α-amylase inhibitory activity in dose-dependent manner. Spinach formulated with 20% lemon juice represents the best source of dietary polyphenols and nitrates with antioxidant and antidiabetic activities that may be used as a functional drink for health and exercise performance improvement.

## Figures and Tables

**Table S1:** pH values of spinach lemon juice formulations

Sample	Original sample	Initial phase	pH Salivary phase	Gastric phase	Intestinal phase
S	(5.06±0.05)^aD^	(6.66±0.04)^aC^	(6.82±0.07)^aB^	(2.24±0.01)^aE^	(7.04±0.02)^aA^
S+L_2_	(4.65±0.01)^bD^	(6.60±0.02)^bC^	(6.65±0.01)^bB^	(2.10±0.02)^bE^	(6.70±0.01)^bA^
S+L_5_	(4.04±0.02)^cD^	(6.13±0.03)^cC^	(6.16±0.02)^cB^	(2.04±0.01)^cE^	(6.64±0.01)^cA^
S+L_10_	(3.83±0.01)^dD^	(5.29±0.02)^dC^	(5.28±0.02)^dB^	(1.94±0.01)^dE^	(6.30±0.01)^dA^
S+L_20_	(3.34±0.01)^eC^	(4.45±0.03)^eB^	(4.39±0.01)^eB^	(1.78±0.02)^eD^	(6.12±0.02)^eA^

Data are presented as mean value±S.D, *N*=4. Different lower case letters indicate significant differences at p≤0.05 in each phase separately. Capital letters indicate significant differences at p≤0.05 among all phases. S=spinach, *φ*(lemon juice)=2, 5, 10 and 20% (L_2_, L_5_, L_10_ and L_20_)

**Table S2:** Pearson’s correlation coefficient between total polyphenols, oxalic acid and nitrates (determined spectrophotometrically), antioxidant activity and pH in digested (salivary phase, gastric phase and intestinal phase) spinach lemon juice formulations

**Salivary phase**	TP/(mg/g)	TF/(mg/g)	TPA/(mg/g)	OA/(mg/g)	Nitrate/(mg/g)	DPPH/%	DPPH/(μmol/g)	FRAP/%	FRAP/(μmol/g)	pH
**TP/(mg/g)**	1.000									
**TF/(mg/g)**	0.502	1.000								
**TPA/(mg/g)**	**0.875**	0.567	1.000							
**OA/(mg/g)**	**0.788**	0.615	**0.943**	1.000						
**Nitrate/(mg/g)**	**0.809**	0.406	**0.833**	**0.766**	1.000					
**DPPH/%**	**0.726**	0.418	**0.922**	**0.935**	**0.718**	1.000				
**DPPH/(μmol/g)**	**0.725**	0.416	**0.922**	**0.934**	**0.718**	**1.000**	1.000			
**FRAP/%**	**0.850**	0.590	**0.967**	**0.901**	**0.731**	**0.868**	**0.868**	1.000		
**FRAP/(μmol/g)**	**0.829**	0.609	**0.975**	**0.986**	**0.769**	**0.940**	**0.939**	**0.961**	1.000	
**pH**	**-0.748**	-0.560	**-0.946**	**-0.966**	**-0.714**	**-0.976**	**-0.973**	**-0.932**	**-0.980**	1.000
**Gastric phase**	TP/(mg/g)	TF/(mg/g)	TPA/(mg/g)	OA/(mg/g)	Nitrate/(mg/g)	DPPH/%	DPPH/(μmol/g)	FRAP/%	FRAP/(μmol/g)	pH
**TP/(mg/g)**	1.000									
**TF/(mg/g)**	**0.795**	1.000								
**TPA/(mg/g)**	**0.735**	**0.906**	1.000							
**OA/(mg/g)**	**0.840**	**0.875**	**0.938**	1.000						
**Nitrate/(mg/g)**	**0.738**	0.448	0.570	**0.770**	1.000					
**DPPH/%**	**0.640**	**0.758**	**0.658**	**0.747**	0.614	1.000				
**DPPH/(μmol/g)**	**0.634**	**0.753**	**0.651**	**0.740**	0.609	**1.000**	1.000			
**FRAP/%**	**0.710**	**0.781**	**0.897**	**0.927**	**0.750**	**0.817**	**0.812**	1.000		
**FRAP/(μmol/g)**	**0.801**	**0.858**	**0.941**	**0.988**	**0.769**	**0.792**	**0.786**	**0.974**	1.000	
**pH**	**-0.788**	**-0.773**	**-0.822**	**-0.940**	**-0.836**	**-0.867**	**-0.863**	**-0.959**	**-0.964**	1.000
**Intestinal phase**	TP/(mg/g)	TF/(mg/g)	TPA/(mg/g)	OA/(mg/g)	Nitrate/(mg/g)	DPPH/%	DPPH/(μmol/g)	FRAP/%	FRAP/(μmol/g)	pH
**TP/(mg/g)**	1.000									
**TF/(mg/g)**	0.350	1.000								
**TPA/(mg/g)**	**0.757**	0.277	1.000							
**OA/(mg/g)**	0.593	0.115	**0.964**	1.000						
**Nitrate/(mg/g)**	**0.761**	0.427	**0.867**	**0.792**	1.000					
**DPPH/%**	0.623	0.262	**0.896**	**0.849**	**0.856**	1.000				
**DPPH/(μmol/g)**	0.620	0.262	**0.890**	**0.841**	**0.854**	**1.000**	1.000			
**FRAP/%**	**0.857**	0.047	**0.815**	**0.719**	0.652	**0.673**	**0.669**	1.000		
**FRAP/(μmol/g)**	**0.742**	0.191	**0.993**	**0.969**	**0.828**	**0.876**	**0.870**	**0.853**	1.000	
**pH**	-0.477	0.151	**-0.714**	**-0.747**	-0.653	**-0.902**	**-0.904**	-0.634	**-0.756**	1.000

Bold values are significant at p≤0.05. TP=total polyphenols, TF=total flavonoids, TPA=total phenolic acids, OA=oxalic acid, DPPH=2,2-diphenyl-1-picrylhydrazyl, FRAP=ferric ion reducing antioxidant power, *φ*(lemon juice)=0, 2, 5, 10 and 20%

**Table S3:** Pearson’s correlation coefficient between of total polyphenols, oxalic acid and nitrates (determined spectrophotometrically), antioxidant activity, antidiabetic activity and pH in predigested spinach lemon juice formulations

Predigested sample	TP/(mg/g)	TF/(mg/g)	TPA/(mg/g)	OA/(mg/g)	Nitrate/(mg/g)	DPPH/%	DPPH/(μmol/g)	FRAP/%	FRAP/(μmol/g)	α-amylase/%	pH
TP/(mg/g)	1.000										
TF/(mg/g)	0.233	1.000									
TPA/(mg/g)	0.616	0.524	1.000								
OA/(mg/g)	**0.670**	0.377	**0.823**	1.000							
Nitrate/(mg/g)	0.440	**0.729**	0.666	**0.693**	1.000						
DPPH/%	0.584	**0.817**	**0.756**	**0.806**	**0.925**	1.000					
DPPH/(μmol/g)	**0.685**	**0.723**	**0.836**	**0.788**	**0.900**	**0.950**	1.000				
FRAP/%	**0.890**	0.319	0.648	**0.835**	**0.667**	**0.748**	**0.766**	1.000			
FRAP/(μmol/g)	**0.850**	0.279	**0.705**	**0.786**	**0.705**	**0.725**	**0.848**	**0.905**	1.000		
α-amylase/%	**0.833**	0.076	**0.712**	**0.730**	0.529	0.548	**0.725**	**0.834**	**0.959**	1.000	
pH	-0.652	**-0.780**	**-0.747**	**-0.738**	**-0.929**	**-0.970**	**-0.978**	**-0.780**	**-0.811**	-0.651	1.000

Bold values are significant at p≤0.05. TP=total polyphenols, TF=total flavonoids, TPA=total phenolic acids, OA=oxalic acid, DPPH=2,2-diphenyl-1-picrylhydrazyl, FRAP=ferric ion reducing antioxidant power, *φ*(lemon juice)=0, 2, 5, 10 and 20%
